# Diagnostic performance of hemozoin-based magneto-optical detection assay and RDT: a prospective observational study

**DOI:** 10.1080/20477724.2025.2551508

**Published:** 2025-09-11

**Authors:** Vishnu Teja Nallapati, Manoj A. R., Sushma Belurkar, Naveenchandra Kulal, Prashanth Bhat, Shama Prasada K, Nitin Gupta, Manjunath H Hande, Priyaleela Thota, David Bell, Kavitha Saravu

**Affiliations:** a Department of Infectious Diseases, Kasturba Medical College, Manipal, Manipal Academy of Higher Education, Manipal, Karnataka,a India; b Department of Pathology, Kasturba Medical College, Manipal, Manipal Academy of Higher Education, Manipal, Karnataka, India; c Department of Health and Family Welfare, Government of Karnataka, Dakshina Kannada District, India; d Department of Health and Family Welfare, Government of Karnataka, Udupi District, India; e Department of Cell and Molecular Biology, Manipal School of Life Sciences, Manipal Academy of Higher Education, Manipal, Karnataka, India; f Department of Medicine, Kasturba Medical College, Manipal, Manipal Academy of Higher Education, Manipal, Karnataka, India; g Hemex Health, Portland, OR, USA

**Keywords:** Hemozoin, magneto-optical detection, rapid diagnostic test, malaria diagnosis, diagnostic performance

## Abstract

Early detection and effective management of malaria are crucial for elimination efforts. Microscopy and rapid diagnostic tests (RDTs) have been the main diagnostic methods for over fifteen years, but they have limitations, especially in cases of low parasite density or deletions of target markers (HRP2/3). This study compares the diagnostic performance of a novel hemozoin-based diagnostic assay (Hz-MOD) with RDTs for detecting malaria in febrile patients in southwestern India. A prospective observational study involved 480 patients screened with Hz-MOD, RDT, microscopy, and nested PCR. Among the samples, 121 were positive by both microscopy and PCR. The sensitivity of Hz-MOD was 94.21% compared to microscopy and 91.74% compared to PCR. For RDTs, sensitivity was 90.91% compared to microscopy and 87.60% compared to PCR. In terms of specificity, Hz-MOD showed 98.61% compared to microscopy and 97.77% compared to PCR, while RDTs had 100% specificity against microscopy and 98.89% against PCR. These results suggest that the hemozoin-based test demonstrates similar sensitivity to RDTs and could serve as an effective screening tool for malaria detection.

## 1. Introduction

Malaria is a vector-borne disease transmitted by infected female *Anopheles* mosquitoes and caused by the genus *Plasmodium*. Five parasite species cause malaria in humans, and two of these species – *Plasmodium falciparum* and *Plasmodium vivax* – pose the greatest threat. The infection is endemic in 83 countries, with approximately 263 million cases and 597,000 deaths reported globally in 2023 [1]. Regardless, proper malaria diagnosis and the species differentiation of *Plasmodium* parasites are still challenging. In 2015, the Global Technical Strategy for Malaria 2016–2030 was adopted by the World Health Organization (WHO) to reduce global malaria incidence and mortality rates by at least 90% by 2030 [2]. This can only be achieved by accurate diagnosis and the appropriate treatment of malaria cases. Even though the available control strategies are effective, they are limited by the ineffectiveness of early diagnostic tools for detection, especially at the low parasite densities required for surveillance in low-transmission settings [3].

As per WHO recommendations, malaria is primarily diagnosed using either microscopy or Rapid Diagnostic Tests (RDT). Even though malaria microscopy is the gold standard technique for diagnosis, it is labor-intensive and highly dependent on the laboratory staff’s degree of expertise. The RDT is an immunochromatographic method in which antibodies are used to capture one or several parasite-specific antigens, such as *Plasmodium falciparum* histidine-rich protein 2 (HRP-2) and *Plasmodium* lactate dehydrogenase (pLDH) or aldolase. More than 200 different RDTs are commercially available and are rapid, easy to perform, and relatively inexpensive. Drawbacks include their inability to quantify parasite density, false positive results due to the persistence of HRP2 in the blood sample for several days after clearance of infection, and false negative results due to the deletion of the target markers (*pfhrp*-2/3 genes) [4,5]. In the past decade, alternative and advanced methods such as Polymerase Chain Reaction (PCR), quantitative PCR (qPCR), real-time PCR (RT PCR), and Loop-mediated isothermal amplification (LAMP) methods have been developed and successfully tested. Although these methods have higher sensitivity and specificity, they are either expensive or require expert personnel. Due to the need for advanced laboratory facilities, highly trained staff, and high costs, these tests cannot be utilized in resource-limited settings [6]. However, the surveillance of low-density parasitemia is crucial for malaria control and elimination, which requires a highly sensitive and field-deployable diagnostic test.

Recently, multiple novel Hz-MOD assays have been developed to detect malaria parasites. These assays are rapid, robust, and sensitive devices that detect hemozoin particles in the blood of malaria-infected patients. Gazelle is one such device, which is a portable, battery-operated point-of-care hemozoin-based malaria diagnostic device with the principle of magneto-optical detection [7–9]. Hence, this study aimed to compare the diagnostic performance of Hz-MOD assays and RDT with microscopy and PCR as reference standards.

## 2. Materials and methods

### 2.1. Study site

A multi-centric prospective observational study was conducted in Kasturba Medical College, Manipal, Malaria Clinic, District Hospital, Udupi, and Malaria Clinic, Govt. Wenlock Hospital Mangalore, between July 2022 and September 2024.

### 2.2. Study settings

Both Udupi (13.3330, 74.7426) and Mangalore (12.8678, 74.8430) are located in the coastal Karnataka region of India, a tropical area characterized by a malaria annual parasite index of 0.01 in Udupi and 0.03 in Mangalore for 2023, with seasonal peaks coinciding with the high rainfall during the monsoon months [10]. The region’s unique combination of urban and rural landscapes, including wetland habitats, provide ideal conditions for mosquito breeding and disease transmission.

### 2.3. Sample size

Assuming sensitivity and specificity to be 90% with 6% of the absolute precision sample size required is 96 malaria-positive and 96 malaria-negative samples. A total of 480 samples were screened to achieve this.

### 2.4. Study procedure

The study was approved by the institutional ethics committee of Kasturba Medical College and Kasturba Hospital (IEC-352/2021) as well as by the technical advisory committee, Directorate of Health & Family Welfare Services, Bengaluru, Karnataka (NHM/SPM/4-Part file/2020–21). The confirmed positive and negative malaria cases (febrile patients) by Microscopy were enrolled after obtaining written informed consent. After recruitment, 2 ml of venous blood samples were collected from all the patients before the administration of antimalarials. Then all the samples were transported to the laboratory at Kasturba Medical College for further analysis.

### 2.5. Laboratory investigations

#### 2.5.1. Malaria microscopy

Thin and thick blood smears were prepared and stained with Leishman’s stain, and two experienced microscopists examined them. A minimum of 200 oil immersion fields were assessed to confirm negative results. The parasite density (parasites/µL) was calculated by dividing the number of asexual parasites counted by the number of white blood cells (WBCs) counted, and then multiplying by an assumed WBC density of 8,000 per µL [11].

#### 2.5.2. RDT

All the samples were tested using the Malaria Ag *P.f/P.v* test (Abbott Bioline™) and interpreted according to the manufacturer’s instructions. This malaria RDT targets detecting histidine-rich protein II (HRP-II) antigen of *P. falciparum* and lactate dehydrogenase (pLDH) of *P. vivax* species in human whole blood.

#### 2.5.3. Hemozoin-based magneto-optical detection assay

A hemozoin-based magneto-optical device, known as Gazelle, developed by Hemex Health, U.S.A., was used for malaria detection. It is a small tabletop reader along with single-use cartridges. Prior to the sample processing, positive and negative controls, along with a blank (empty cartridge), were analyzed for quality control (QC). All the samples were processed as per the manufacturer’s instructions. Briefly, 65 µL of the assay buffer was added to the test cartridge (a disposable single-use item), followed by the addition of 30 µL of whole blood, and then the cartridge was loaded into the device for interpretation. In three minutes, the device displayed the results, and those were recorded.

#### 2.5.4. Molecular diagnosis

DNA extraction was done using the QIAamp DNA Blood Mini Kit (Qiagen, Germany) and eluted with 100 μL of elution buffer. The DNA was amplified by nested PCR to detect malaria parasites using genus/species-specific primers targeting the 18S rRNA gene following the protocol of Snounou et al. [12]. All reactions were carried out in a final volume of 25 µL containing 2 mM MgCl_2_, 50 mM KCl, 10 mM Tris – HCl pH 8.3, 125 µM of each dNTPs, 250 nM of each oligonucleotide primer, 0.5 units of Taq DNA polymerase, and 2 µL of template DNA. The amplification of amplicons were visualized on a 1.5% agarose gel with EtBr staining.

### 2.6. Statistical analysis

Two-by-two contingency tables were constructed to include True Positives (TP), True Negatives (TN), False Positives (FP), and False Negatives (FN) for each index test, as well as various combinations of the components of the combination tests, compared to the reference tests. We analyzed the results for sensitivity, specificity, positive and negative likelihood ratios, and diagnostic odds ratio (DOR). Likelihood ratios for positive (LR+) and negative (LR−) test results were considered good when LR+ was > 10, and LR− < 0.1 [13]. The higher DOR indicates better test performance, with values >1 suggesting the test discriminates well between diseased and non-diseased individuals. For example, a DOR of 10 means the odds of a positive test are 10 times higher in those with the condition [14]. Diagnostic accuracy was assessed through receiver-operating characteristics (ROC) curves, and the area under the curve (AUC) was interpreted as follows: 0.9–1.0, excellent; 0.8–0.9, very good; 0.7–0.8, good; 0.6–0.7, sufficient; 0.5–0.6, bad; <0.5, test not useful [15]. The data analysis was performed by using SPSS version 20 and MedCalc’s software [16].

## 3. Results

**Figure 1.**
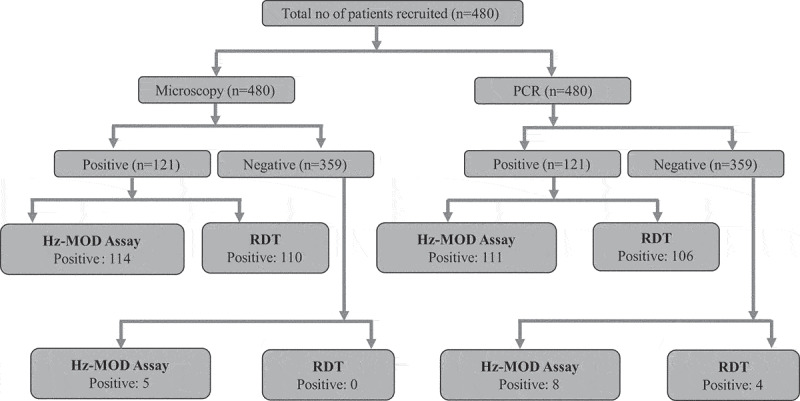
Flow chart illustrating the diagnostic workflow and test results for the 480 patients recruited in the study.

A total of 480 febrile patients were enrolled, with 121 confirmed positive by microscopy and 359 confirmed negative for malaria (Figure 1). However, we observed discrepancies between the microscopy and PCR results. Of the total samples, 68.5% were males and 31.5% were females; their mean age was 38.07 ± 16.85 years. Among the malaria-positive cases, 105 (86.77%) were *P. vivax*, 11 (9.09%) were *P. falciparum*, and 5 (4.13%) were mixed infections. The median parasite density of the samples was 3388 parasites/μL (interquartile range [IQR]: 988 parasites/μL, 8827 parasites/μL). In *P. vivax* samples, the median parasite density was 2456 parasites/μL (IQR: 917 parasites/μL, 7484 parasites/μL), whereas it was 8294 parasites/μL (IQR: 1751 parasites/μL 12,088 parasites/μL) in *P. falciparum* samples. When evaluating diagnostic performance, out of 121 microscopy-positive malaria samples, 114 tested positive by Hz-MOD, 110 by RDT, and 118 by PCR (Figure 1). Conversely, among the 121 PCR-positive malaria samples, 111 tested positive by Hz-MOD, 106 by RDT, and 118 by microscopy (Figure 1).

### 3.1. Diagnostic accuracy of index tests as compared to microscopy

**Figure 2.**
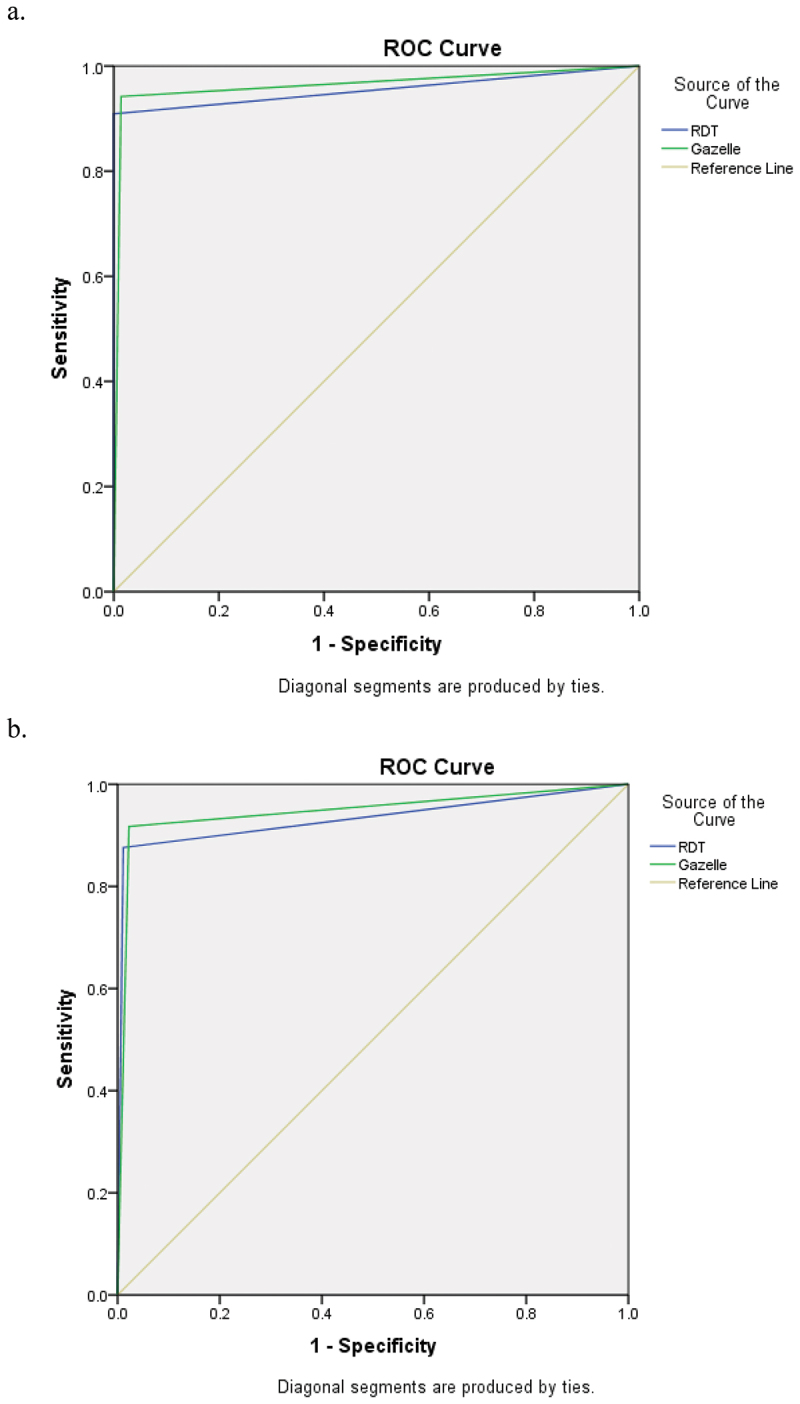
ROC curves for Hz-MOD and RDT compared to (a) microscopy and (b) PCR.

The sensitivity and specificity of the Hz-MOD assay were 94.21% (95% CI: 88.44–97.64) and 98.61% (95% CI: 96.78–99.55), while the RDT sensitivity and specificity were 90.91% (95% CI: 84.32–95.37) and 100% (95% CI: 98.98–100), respectively (Table 1). The pooled estimates for the LR+, LR−, and DOR for hemozoin-based diagnostic assay were 67.65 (95% CI: 28.30–161.71), 0.06 (95% CI: 0.03–0.12), and 1153.03 (95% CI: 358.97–3703.57), respectively, and the overall LR− and DOR for RDT were 0.09 (95% CI: 0.05–0.16), and 6908.65 (95% CI: 403.85–118186.58), respectively (Table 1). However, LR+ for RDTs was not available. The AUC for Hz-MOD and RDT were 0.964 (95% CI: 0.939–0.989) and 0.955 (95% CI: 0.924–0.985), respectively (Figure 2).

**Table 1.** Diagnostic performance of Hz-MOD and RDT for diagnosis of malaria.

Reference standard	Index tests	Sensitivity % (95%CI)	Specificity % (95%CI)	Positive Likelihood Ratio (95%CI)	Negative Likelihood Ratio (95%CI)	Diagnostic Accuracy (95%CI)	Diagnostic Odds Ratio (95%CI)	P value
Microscopy as the reference standard	Hz-based diagnostic assay	94.21% (88.44–97.64)	98.61 (96.78–99.55)	67.65 (28.30–161.71)	0.06 (0.03–0.12)	97.50 (95.67–98.70)	1153.02 (358.97–3703.57)	< 0.0001
	RDT	90.91 (84.32–95.37)	100 (98.98–100)	–	0.09 (0.05–0.16)	97.71 (96.94–98.85)	6908.65 (403.84–118186.58)	< 0.0001
PCR as the reference standard	Hz-based diagnostic assay	91.74 (85.33–95.97)	97.77 (95.66–99.03)	41.17 (20.70–81.85)	0.08 (0.05–0.15)	96.25 (94.14–97.76)	487.01 (187.61–1264.18)	< 0.0001
	RDT	87.60 (80.38–92.89)	98.89 (97.17–99.70)	78.62 (29.60–208.83)	0.13 (0.08–0.20)	96.04 (93.89–97.60)	627.16 (203.79–1930.04)	< 0.0001

Abbreviations: TP-true positive; FP-false positive; FN-false negative; TN-true negative; PCR – polymerase chain reaction; RDT – rapid diagnostic tests; Hz-MOD – hemozoin-based magneto-optical detection assay.

In *P. vivax* samples, the sensitivity and specificity of the Hz-MOD assay were 94.29% (95% CI: 87.98–97.87) and 98.61% (95% CI: 96.78–99.55), respectively, while the RDT sensitivity and specificity were 90.48% (95% CI: 83.18–95.34) and 100% (95% CI: 98.98–100). In contrast, in *P. falciparum* samples, the sensitivity and specificity of the Hz-MOD assay were 90.91% (95% CI: 58.72–99.77) and 98.61% (95% CI: 96.78–99.55), while the RDT sensitivity and specificity were both 100% (95% CI: 71.51–100.00) and 100% (95% CI: 98.98–100), respectively (Table 2).

**Table 2.** Comparison of sensitivity and specificity of Hz-MOD and RDT for the detection of *P. vivax* and *P. falciparum.*

		Microscopy as a reference test		PCR as a reference test	
		Sensitivity % (95% CI)	Specificity % (95% CI)	Sensitivity % (95% CI)	Specificity % (95% CI)
Hz-MOD	In *P. vivax* samples	94.29 (87.98–97.87)	98.61 (96.78–99.55)	91.51 (84.49–96.04)	97.77 (95.66–99.03)
	In *P. falciparum* samples	90.91 (58.72–99.77)	98.61 (96.78–99.55)	91.67 (61.52–99.79)	97.77 (95.66–99.03)
RDT	In *P. vivax* samples	90.48 (83.18–95.34)	100 (98.98–100)	95.88 (89.78–98.87)	100 (98.98–100)
	In *P. falciparum* samples	100 (71.51–100.00)	100 (98.98–100)	100 (73.54–100.00)	100 (98.98–100)

Abbreviations: PCR – polymerase chain reaction; RDT – rapid diagnostic tests; Hz-MOD – hemozoin-based magneto-optical detection assay.

For samples with high parasite density (> 200 parasites/μL), Hz-MOD showed a sensitivity of 95.54% (95% CI: 89.89–98.53), surpassing RDT, which had a sensitivity of 91.07% (95% CI: 84.19–95.64). For samples with low parasite density (< 200 parasites/μL), Hz-MOD demonstrated a sensitivity of 100% (95% CI: 66.37–100), while RDT showed a sensitivity of 88.89% (95% CI: 51.75–99.72%).

### 3.2. Diagnostic accuracy of index tests as compared to PCR

The sensitivity and specificity of the Hz-MOD assay were 91.74% (95% CI: 85.33–95.97) and 97.77% (95% CI: 95.66–99.03), while the RDT sensitivity and specificity were 87.60% (95% CI: 80.38–92.89) and 98.89% (95% CI: 97.17–99.70), respectively (Table 1). The pooled estimates for the LR+, LR−, and DOR for hemozoin-based diagnostic assay were 41.17 (95% CI: 20.70–81.85), 0.08 (95% CI: 0.05–0.15), and 487.01 (95% CI: 187.62–1264.19), respectively, and the overall LR+, LR−, and DOR for RDT were 78.62 (95% CI: 29.60–208.83), 0.13 (95% CI: 0.08–0.20), and 627.17 (95% CI: 203.79–1930.04), respectively (Table 1). The AUC for Hz-MOD and RDT were 0.948 (95% CI: 0.918–0.977) and 0.932 (95% CI: 0.897–0.968), respectively (Figure 2).

In *P. vivax* samples, the sensitivity and specificity of the Hz-MOD assay were 91.51% (95% CI: 84.49–96.04) and 97.77% (95% CI: 95.66–99.03), respectively, while the RDT sensitivity and specificity were 95.88% (95% CI: 89.78–98.87) and 100% (95% CI: 98.98–100). In contrast, in *P. falciparum* samples, the sensitivity and specificity of the Hz-MOD assay were 91.67% (95% CI: 61.52–99.79) and 97.77% (95% CI: 95.66–99.03), while the RDT sensitivity and specificity were both 100% (95% CI: 73.54–100.00) and 100% (95% CI: 98.98–100), respectively (Table 2).

## 4. Discussion

The Global Technical Strategy for Malaria 2016–2030 aims to reduce malaria incidence and mortality at least by 90% by 2030 [2]. Early detection and management of malaria is crucial; however, detecting low-density parasitemia is challenging. Furthermore, many countries are progressing from malaria control to elimination. Therefore, accurate, reliable, and field-deployable rapid diagnostic approaches are essential to achieve this goal. Thus, in this study, we compared the diagnostic performance of one such Hz-MOD assay called Gazelle by Hemex Health, U.S.A. [17,18], with the malaria RDT (PfHRP2/pLDH).

In our analysis, we observed that the overall diagnostic accuracy of Hz-MOD and RDT compared to Microscopy were 97.5% (95% CI: 95.6–98.7) and 97.7% (95% CI: 95% CI: 96.9–98.8). Compared to PCR, the diagnostic accuracy of Hz-MOD and RDT were 96.25% (95% CI: 94.1–97.7) and 96.0% (95% CI: 93.8–97.6). These results were similar to the study conducted by Kumar et al., where the diagnostic accuracy of Hz-MOD and RDT were 96.9% (95% CI: 94.1–98.7) and 93.5% (95% CI: 89.8–96.2) compared to microscopy, whereas it was 95.4% (95% CI: 92.1–97.6) and 94.3% (95% CI: 90.7–96.8) compared to PCR [8].

In our study, we assessed the sensitivity of diagnostic assays by parasite density. The Hz-MOD assay successfully detected 69 parasites/µL in clinical samples of *P. vivax*. Notably, these results suggest that Hz-MOD may offer superior performance, particularly in detecting low-density infections where conventional RDTs are less reliable. However, the sample size for the low parasite density group was limited (*n* = 9), resulting in wide confidence intervals and reduced statistical power. This limitation may affect the precision and generalizability of our sensitivity estimates, especially for low-density parasitemia cases. Therefore, larger studies are needed to assess diagnostic performance in low-transmission or pre-elimination settings, where low-density infections are more prevalent and present a significant challenge for malaria elimination efforts.

Our findings are consistent with reports from other endemic regions, such as Latin America, where the diagnostic sensitivity of both Hz-MOD and RDTs tends to be lower in low-transmission settings characterized by a higher prevalence of low-density infections. This pattern is evident in studies from the Brazilian Amazon, where the diagnostic accuracy of Hz-MOD and RDT for *P. vivax* malaria was high when compared to microscopy (98.2% and 92.4%, respectively), but substantially lower when compared to PCR (82.3% and 76.5%, respectively) [9]. Similar trends were observed in another study from the same region, with Hz-MOD and RDT reporting the diagnostic accuracy of Hz-MOD and RDT as 96.7% and 94.0% compared to microscopy, while it was 92.9% and 90.2% compared to PCR [19].

Furthermore, the Hz-MOD demonstrated a sensitivity of 84.6% in *P. knowlesi* cases (*n* = 26) with ≤200 parasites/µL, with the lowest parasitemia detected being 18/µL [20]. Moreover, previous studies have reported the limit of detection as low as 50 parasites/µL in the cultured *P. falciparum* isolates with an accuracy of 95%. In contrast, in clinical samples of patients infected with *P. vivax* malaria, the limit of detection was found to be 35 parasites/µL, with an accuracy of 100% [8]. Various studies that evaluated the diagnostic performance of the Hz-MOD (Gazelle) assay and RDT have reported diagnostic accuracy ranges of 95.1%−99.6% for the Hz-MOD and 92.4%−100% for RDT when compared to microscopy. In contrast, the diagnostic accuracies of Hz-MOD and RDT ranges were observed to be 85.1%–99.6% and 76.4%–100% compared to PCR (Table 3) [8,9,19–22].

**Table 3.** Comparison of diagnostic performance between Hz-MOD and RDT.

Author with reference	Year of the study	Study population	Location of the study	Microscopy as a reference test				PCR as a reference test			
				Hz-MOD (Gazelle)		Malaria RDT		Hz-MOD (Gazelle)		Malaria RDT	
				Sensitivity % (95%CI)	Specificity % (95%CI)	Sensitivity % (95%CI)	Specificity % (95%CI)	Sensitivity % (95%CI)	Specificity % (95%CI)	Sensitivity % (95%CI)	Specificity % (95%CI)
Kumar et al. [8],	2020	Malaria infections	ICMR-NIRTH, Jagdalpur, Chhattisgarh, India	97.6 (87.4–99.9)	96.8 (93.6–98.7)	100.0 (91.6–100.0)	92.3 (87.9–95.4)	82.1 (69.6–91.1)	99.0 (96.5–99.9)	89.3 (78.1–96.0)	95.6 (91.9–98.0)
De Melo, G.C., et al, [9]	2021	*P. vivax* samples	Western Brazilian Amazon	96.1 (91.3–98.3)	100 (97.4–100)	83.8 (76.3–89.7)	100 (97.51–100)	72.1 (65.0–78.3)	99.0 (94.8–99.9)	62.79 (55.11–70.03)	99.04 (94.7–99.9)
Valdivia HO et al. [19],	2021	*P. vivax* samples	Peruvian Amazon basin	96.15 (89.1–99.2)	98.04 (93.10–99.7)	89.74 (80.7–95.4)	100 (96.45–100)	88.2 (79.4–94.2)	97.9 (92.6–99.7)	82.4 (72.6–89.8)	100 (96.2–100.0)
Fernando, D et al. [21],	2022	Malaria infections	AMC Headquarters, Colombo, Sri Lanka.	77.8 (40.0–97.2)	100.0 (99.2–100.0)	100.0 (66.3–100.0)	100.0 (99.1–100.0)	77.8 (40.0–97.2)	100.0 (99.2–100.0)	100.0 (66.3–100.0)	100.00 (99.1–100.0)
Fontecha, G. et al. [22],	2022	Malaria infections	Department of Gracias a Dios in the Moskitia region, Eastern Honduras	92.00 (80.7–97.7)	98.82 (95.8–99.8)	–	–	59.7 (57.9–61.6)	98.6 (98.2–99.0)	–	–
Tan, A.F. et al. [20],	2023	*P. knowlesi* samples	Ranau District Hospital in Sabah, Malaysia Menzies School of Health Research, Australia	94.2 (90.5–96.8)	100.0 (91.9–100.0)	–	–	92.71 (88.7–95.6)	100.00 (91.1–100.0)	–	–

The variation in diagnostic accuracy across different epidemiological contexts is likely multifactorial. One key factor is the higher proportion of asymptomatic or sub-patent infections in low-transmission settings, which often present with parasite densities below the detection thresholds of both Hz-MOD and RDTs [23,24]. Additionally, differences in host immunity may allow infections to persist at lower densities for longer periods, further complicating detection [25]. Variations in parasite biology, such as differences in hemozoin production or antigen expression, may also influence assay performance [26,27]. Finally, methodological factors, including the choice of reference standard and sample handling, can impact measured diagnostic accuracy [28]. Collectively, these considerations highlight the need for context-specific validation and optimization of malaria diagnostic tools, particularly as control programs shift focus toward elimination and the detection of low-density infections becomes increasingly critical [29].

In this study, we found that Hz-MOD (Gazelle) demonstrated promising sensitivity, particularly when compared to the RDT, excelling in the detection of symptomatic malaria infections. This suggests that the Hz-MOD assay may address some of the inherent limitations associated with RDTs. Notably, the false positivity rate of Hz-MOD was 1.39%, which is well within the current WHO selection criteria for RDT procurement (< 10%) [30]. One of the most significant advantages of Hz-MOD (Gazelle) is its remarkably quick turnaround time with a processing time of just three minutes, it far outperforms traditional microscopy (30 minutes), PCR (7–8 hours), and even RDT (15–20 min) making it a valuable diagnostic tool for fieldwork, where rapid and accurate diagnosis is essential (Table 4). Such rapid detection could potentially improve malaria management in resource-limited settings, where speed and accessibility are crucial.

**Table 4.** Comparative cost analysis of Hz-MOD and Conventional malaria diagnostic assays.

Diagnostic Method	Cost per Test (USD)	Cost Components	Time per Test	Advantages	Disadvantages	Reference
Hz-MOD (Gazelle)	$2.18–$2.36	Device amortization, materials, and minimal labor	~3 min	Sensitive; Rapid; Cost effective	Cannot differentiate species	[31]
RDT (Rapid Diagnostic Test)	$1.00–$2.19	Materials ($1.00–$1.51), labor ($0.68)	~15–20 min	Rapid, easy to perform, and inexpensive	Unable to quantify parasitemia; Absence of HRP 2 leads to false negative results	[30,32]
Microscopy	$6.98	Materials ($0.18), labor ($6.80)	~30–60 min	Gold standard test and high specificity	Labor-intensive and slow, and limited sensitivity at low parasitemia	[32]
PCR	$20–$60	Reagents, skilled labor, and equipment	7–8 hours	Highest sensitivity/specificity	Costly, slow, and requires lab infrastructure; not suitable for field use	[33,34]

The estimated cost covers reagents and supplies for DNA extraction and slide preparation, including equipment and labor costs.

However, despite its promising performance, the Hz-MOD (Gazelle) assay has limitations. It cannot differentiate between malaria species, which is a critical shortcoming in areas where both *P. vivax* and *P. falciparum* co-exist. Consequently, any positive result obtained from Hz-MOD would require further species-specific testing via microscopy, which increases both time and cost. Additionally, hemozoin-based assays like Hz-MOD (Gazelle) are prone to false negatives in early or low-density infections due to insufficient hemozoin production and false positives from residual hemozoin from prior infections or technical artifacts [22,35,36]. These factors can reduce diagnostic accuracy. Although only minimal training is required to operate the device, the initial cost of the Hz-MOD (Gazelle) remains a significant financial consideration. Given these factors, there is a clear need for an updated version of the Hz-MOD (Gazelle) that can distinguish between different malaria species while also enhancing sensitivity and specificity. Such improvements could make Hz-MOD an even more effective diagnostic tool, potentially transforming malaria detection across diverse settings.

## 5. Limitations

The study was conducted in a region with a high prevalence of *P. vivax* malaria and relatively fewer cases of *P. falciparum*, and analysis would benefit from a larger sample size.

## 6. Conclusion

Hz-MOD (Gazelle) assay has shown better sensitivity than RDT in detecting *Plasmodium* infections with similar diagnostic accuracy. This suggests that the Hz-MOD assay could be an alternative for malaria screening. However, the Hz-MOD assay cannot distinguish between malaria species. Hence, to fully understand the true potential of the Hz-MOD assay, it needs an evaluation on a large number of febrile patients with a modified version of the assay which can differentiate among the species across various malaria settings.
